# Clinical management of the first ASCUS report in Chile. Prospective single-cohort study: Manejo clínico do primeiro relatório ASCUS no Chile. Estudo prospectivo de coorte única

**DOI:** 10.1590/1516-3180.2014.9142511

**Published:** 2015-10-09

**Authors:** Fanny López-Alegría, Orlando Quezada Poblete, Dino Soares De Lorenzi, Juan Carlos Sepúlveda Oyanedel

**Affiliations:** I PhD. Associate Professor, School of Nursing, Universidad Andres Bello, Santiago, Chile.; II MT. Medical Technologist, Cytology Laboratory, Complejo Asistencial Barros Luco, Santiago, Chile.; III MD, PhD. Adjunct Professor, Department of Obstetrics and Gynecology, Universidade de Caxias do Sul (UCS), Caxias do Sul, Rio Grande do Sul, Brazil.; IV PhD. Sociologist, Quantitative Studies and Public Opinion Program, Universidad de Santiago de Chile, Santiago, Chile.

**Keywords:** Papanicolaou test, Uterine cervical dysplasia, Diagnosis, Practice guidelines as topic, Follow-up studies.

## Abstract

**CONTEXT AND OBJECTIVE:**

Worldwide, there is no single strategy for optimal management of patients with ASCUS (atypical squamous cells of undetermined significance) cytology reports. The objective of this study was to determine the kind of clinical management conducted among women with a first ASCUS Pap smear report.

**DESIGN AND SETTING:**

Prospective single cohort study at a cervical pathology unit in Santiago, Chile. METHODS: This was an epidemiological, descriptive, observational and quantitative follow-up study on a cohort of women with ASCUS cytological reports.

**RESULTS:**

In the screening phase, 92,001 cervical cytological smears were collected in primary healthcare clinics. In the diagnostic phase, all women with a first ASCUS report were selected (n = 446). These women were asked to undergo the Pap test again and it was found that 301 women had normal results, 62 women had abnormal results and 83 did not repeat the test. In the diagnostic confirmation phase, the 62 women with abnormal results underwent colposcopy and, from these results, 58 of them underwent a biopsy. The results from the biopsies showed that 16 women had negative histological reports, 13 had CIN 1 and 29 had CIN 2+. In the treatment phase, the 42 women with lesions underwent a variety of treatments, according to the type of lesion. In the post-treatment phase, cytological and colposcopic monitoring was instituted.

**CONCLUSION:**

The clinical management consisted of traditional management of screening, diagnosis, diagnostic confirmation, treatment and post-treatment monitoring.

## RESUMO

**CONTEXTO E OBJETIVO:**

Mundialmente, não existe estratégia única para o gerenciamento ideal de pacientes com laudos de citologia ASCUS (*atypical squamous cells of undetermined significance*). O objetivo do estudo foi determinar o tipo de gerenciamento clínico realizado em mulheres com o primeiro laudo de Papanicolaou ASCUS.

**DESENHO E LOCAL:**

Estudo prospectivo de coorte única em uma unidade de patologia cervical em Santiago, Chile.

**MÉTODOS:**

Estudo epidemiológico, descritivo, observacional e quantitativo de seguimento de um grupo de mulheres com laudos de citologia ASCUS.

**RESULTADOS:**

Na fase de rastreamento, 92.001 relatórios de citologia cervical foram coletados nos ambulatórios da rede Pública de saúde. Na fase de diagnóstico, foi selecionada a totalidade de mulheres com relatórios citológicos de ASCUS (n = 446). Essas mulheres foram submetidas a repetição do exame de Papanicolaou, obtendo-se 301 mulheres com resultados normais, 62 mulheres com resultados anormais e 83 que não repetiram o exame. Na fase de confirmação de diagnóstico, as 62 mulheres com resultados anormais foram submetidas a colposcopia e, considerando-se os resultados obtidos, 58 foram submetidas a biópsia. Os resultados das biópsias mostraram 16 mulheres com laudos histológicos negativos, 13 com NIC 1 e 29 com NIC 2 +. Na fase terapêutica, as 42 mulheres com lesões foram submetidas a diversos tratamentos de acordo com o tipo de lesão. Para a fase pós-tratamento, foi instituído um sistema de monitoramento citocolposcópico.

**CONCLUSÃO:**

As condutas clúnicas tiveram gestão tradicional de rastreamento, diagnóstico, confirmação diagnóstica, tratamento e acompanhamento pós-tratamento.

## INTRODUCTION

Atypical squamous cells of undetermined significance (ASCUS) are the most prevalent abnormality in cervical cytological evaluations. This is a category of uncertain morphology and is at the limit between normal and abnormal cytological interpretations.^1^ ^2^The clinical significance of ASCUS is considered to be indeterminate, which means that there is controversy or debate regarding its clinical management, given that there is no single choice for optimal treatment.^3^ ^4^

In Chile, the Ministry of Health (Minsal) has developed clinical guidelines that contain two clinical decision diagrams called the "algorithm for conveying the first atypical Pap test according to Bethesda 2001 classification, to a specialist or cervical pathology unit (CPU)" and the "algorithm for diagnostic confirmation".^5^ ^6 ^These diagrams contain the steps for diagnostic confirmation and treatment of cervical atypia and are based on the first consensus guidelines for management of these cervical atypia, published by Wright et al.,^7^ which were based on the multicenter study titled Atypical Squamous Cells of Undetermined Significance/Low-grade Squamous Intraepithelial Lesion Triage Study (ALTS).^8^

In Chile there is a paucity of scientific evidence on the clinical management of women with cervical atypia.^9^ In addition, the national conveyance algorithm and national diagnostic confirmation algorithm are supported by evidence derived from English-speaking countries.^10^ ^13^ This situation makes it appropriate and relevant to conduct studies that generate current and local epidemiological evidence for managing atypical cervical cytology. This challenge is consistent with the objectives set by Minsal, which has the goal of generating clinical algorithms supported by national scientific evidence.^14^

### OBJECTIVE

The objective of this study was to determine the kind of clinical management performed among females with a first ASCUS report, diagnosed between 2008 and 2009 and followed up at the Cervical Pathology Unit of the Barros Luco Hospital (primary care hospital base) within the Southern Metropolitan Healthcare Area of Santiago, Chile.

## METHODS

An epidemiological study was performed consisting of descriptive, prospective, observational and quantitative follow-up was conducted on a cohort of women with ASCUS cytology reports who formed part of the National Program for Research and Control of Cervical Cancer in Chile. 

From 2008 to 2009, 92,001 exfoliative cervical cytological smears were collected by professionals at primary healthcare clinics (PHCs) in the southern metropolitan area of Santiago, Chile (Table 1). These smears were processed by means of the conventional technique of Pap sampling and were classified in accordance with the national classification system, which is equivalent to that of Bethesda 2001 as detailed below. This was done in the Cytology Laboratory of the Pathology Department of the Barros Luco Hospital in Santiago, Chile.^15^ ^16^ Subsequently, these records were filed in the Diagnostics Archive of Minsal's Cyto-Expert System (a cytological-histological database). At this time, all the ASCUS reports were selected (n = 555) (Table 1). 

Selection criteria were applied to these patients, so as to select patients without previous uterine pathological conditions, without previous cervical procedures and with normal Pap results over a three-year period prior to the beginning of the study. After making this selection, a cohort of 446 women with ASCUS reports was obtained and these women were monitored for a period of three years, or until resolution of the case or until loss from the follow up. They were treated at the Cervical Pathology Unit of the Barros Luco Hospital.

For data gathering, the Cyto-Expert database and the patient's clinical records were used. 

The variables studied included the women's ages at the time when ASCUS was identified; the type and number of cytological, colposcopic and histological results; and treatments instituted.

The national classification was used for coding the cytological variable. This is equivalent to that of Bethesda 2001 and uses the following categories: negative for intraepithelial lesion or malignancy (Neg); atypical squamous cells (ASC); atypical squamous cells of undetermined significance (ASCUS); low-grade squamous intraepithelial lesion (LSIL); and high-grade squamous intraepithelial lesion (HSIL).

**Table 1:** Screening phase: exfoliative cervical cytological examinations from the primary care health services of Santiago, Chile, 2008 - 2009

Cytology	Result	Number	%
Negative cytology	Satisfactory	79,521	86.43
	Less than optimal	4,062	4.41
	Inadequate	6,679	7.26
Atypical			
Atypical squamous cells of undetermined significance (ASCUS)		555	0.60
Atypical squamous cells cannot exclude high-grade SIL (ASC-H)		150	0.16
Atypical glandular cells of undetermined significance (AGC-US)		41	0.04
Atypical glandular cells suggestive of adenocarcinoma in situ		13	0.01
Positive cytology	Low-grade Pap	486	0.53
	High-grade Pap	444	0.48
	Invasive cancer	50	0.05
Total		92,001	99.94

The following definitions were used in coding the colposcopic variable. Normal colposcopy (Neg) was defined as negative colposcopic findings of intraepithelial lesions; colposcopic intraepithelial lesions (IEL) were defined as colposcopic findings showing a squamous cervical lesion; colposcopic human papillomavirus (HPV) was defined as presence of lesions in the cervix probably caused by HPV; squamous cell carcinoma (SCC) was defined as the presence of visual characteristics of this disease; and undetermined colposcopy was defined as uncertainty in the definition of the diagnosis. In addition, we included the variable of colposcopic HPV + IEL, defined as presence of both of these conditions.

Finally, cervical intraepithelial neoplasia (CIN) codes were used in coding the histological variable. These were classified as: negative for intraepithelial lesion or malignancy (Neg); mild cervical intraepithelial neoplasia (CIN 1); moderate cervical intraepithelial neoplasia (CIN 2); severe cervical intraepithelial neoplasia (CIN 3); carcinoma "in situ" (CIS); and squamous cell carcinoma (SCC).

Descriptive statistics were used for data analysis. These identified the number, type and outcome of the procedures performed, and also the time intervals between the first atypical ASCUS result and i) diagnosis; ii) diagnostic confirmation; iii) treatment; and iv) post-treatment evaluation.

In order to present the information obtained in the investigation graphically, a tree diagram of conditional probability was constructed. In this diagram, a circle was used to represent cervical cytological analysis, an octagon for losses from the followup, a square for colposcopic analysis and a box for biopsy. In this design, the number indicated the quantity of patients and the classifications, the examination results or the clinical or surgical procedures.

This study was approved by the Andres Bello University Ethics Committee and the Scientific Ethics Committee of the Southern Metropolitan Healthcare Service in Santiago, Chile.

## RESULTS

The study population consisted of 446 women with ASCUS cytology reports. The average age of the women was 37.74 years, with a minimum age of 17 and maximum age of 88. The clinical management performed was based on the national clinical algorithms.

The follow-up was presented in five phases: screening, diagnosis, diagnostic confirmation, treatment and post-treatment evaluation.

In the first phase (screening), 92,001 Pap tests were collected. A result consisting of ASCUS was found in 0.60% of this population (n = 555) (Table 1). A cohort of 446 women was obtained from this group by applying the selection criteria of our study.

**Figure 1:**
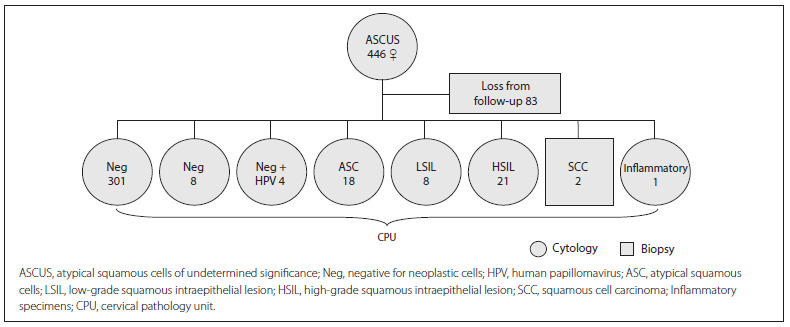
Diagnostic stage: repeated Pap smears in women with first ASCUS reports.

In the second phase (diagnosis), atypical Pap tests should be repeated at the same PHC within six months, in accordance with the national algorithms. Out of the total of 446 women, 83 did not come back for cytological smear collection within a period of one year and were considered to have been lost from the follow-up. The remaining women obtained the following results: i) 301 patients were negative for intraepithelial lesions or malignancy (Neg) and, in accordance with the national algorithms, continued with the scheme of repeating Pap screening every three years; and ii) 62 women whose cytological findings were divided into: Neg (n = 8), Neg + HPV (n = 4), ASC (n = 8), LSIL (n = 8), HSIL (n = 21), inadequate smear with inflammatory cytological findings (n = 1) and SCC (n = 2). This last result was obtained through biopsy. These 62 women with cytohistological alterations and/or clinical symptoms were referred to the CPU to elucidate the cytological diagnosis (Figure 1).

**Figure 2:**
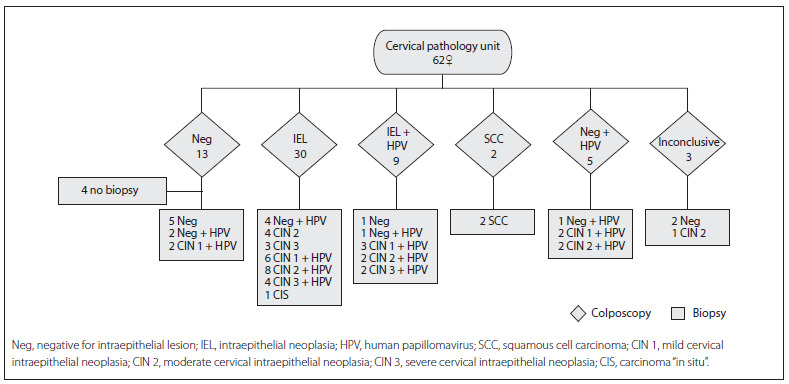
Diagnostic confirmation stage: histological and colposcopic follow-up results in women with ASCUS.

In the third phase (diagnostic confirmation), two procedures performed at the CPU were used: colposcopy and biopsy. A colposcopic examination was carried out on 62 women and showed the following results: negative colposcopy (Neg) (n = 13), IEL (n = 30), IEL + HPV (n = 9), SCC (n = 2), Neg + HPV (n = 5) and undetermined colposcopic findings (n = 3). Given the results observed, biopsies were carried out on 58 women during the same procedure, at the same time. Four women presented normal colposcopic results and therefore did not undergo this procedure. In most of the women, negative colposcopic findings (n = 13) were correlated with negative histological findings. The result was confirmed in the cases of seven women (negative for cervical intraepithelial neoplasia) and two others were found to have CIN 1. HPV was present in the remaining four women (Figure 2).

Among the patients diagnosed with IEL (n = 39), the colposcopic results coincided with the histological results in 33 cases, and these results were: CIN 1 (n = 9), CIN 2 (n = 14), CIN 3 (n = 9) and CIS (n = 1). The remaining six were negative for intraepithelial neoplasia (Neg). 

The patients diagnosed with squamous cell carcinoma (n = 2) were confirmed histologically to have stage IB2 cervical cancer (SCC).

The patients whose colposcopy was defined as Neg + HPV (n = 5) received confirmation of the presence of HPV from the histological result.

Finally, the cases of undetermined colposcopic findings (n = 3) were defined by means of histological examinations, such that two cases were negative for lesions (Neg) and one presented CIN 1.

In summary, the results from the 58 biopsies performed were divided into: negative for intraepithelial lesions (Neg, n = 16), low-grade lesion (CIN 1, n = 13) and high-grade lesion (CIN 2 +, n = 29). Presence of HPV was detected in 39 histological examinations (Figure 2).

In the fourth phase (treatment), some negative biopsy results (n = 12) and the CIN 1 + HPV result of a pregnant woman were excluded, giving a total of 45 women who were under treatment. For therapeutic resolution of these cases, one of the following procedures was used: cervical cryosurgery, surgical conization (CKC), hysterectomy (HT) or chemotherapy and radiotherapy (CT + RT).

 Patients with mild lesions (CIN 1) underwent cryotherapy (n = 4) and needle biopsy (n = 7). Those with moderate and severe lesions (CIN 2/3) underwent cold-knife cone biopsy (n = 26) and hysterectomy (n = 1). The patient with CIS (n = 1) underwent hysterectomy and the patients with stage IB2 cervical cancer (n = 2) underwent chemotherapy and radiotherapy. One patient presented CIN 1 + HPV contained in a cervical polyp (n = 1), which was removed (Figure 3).

Finally, in the fifth phase (post-treatment follow-up), three procedures were carried out: colposcopy, cytological sample collection and histological sample collection (if necessary). 

In addition, a general physical and gynecological examination was performed. These procedures were carried out over a period of approximately three years, which was a requirement in order to discharge the patients from the algorithm and to return them to their corresponding PHC.

The average time interval between the collection of the first ASCUS report and the second phase (diagnosis through a repetition of atypical Pap tests) was 194.34 days. The time from this first report to the third phase (diagnostic confirmation) was 402.89 days; the time to the fourth phase (treatment) was 536.75 days; and the time to the fifth phase (post-treatment follow-up) was 841.51 days.

**Figure 4:**
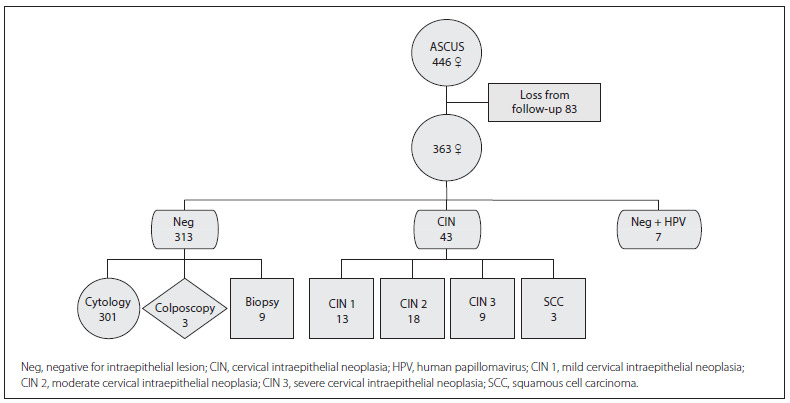
Follow-up results among women with ASCUS, and diagnostic confirmation procedure.

At the end of the follow-up period, the cohort of 446 female with a first ASCUS report achieved the following results (excluding the 83 women who had been lost from the follow-up): negative for neoplasia (n = 313) (86.2%); and negative for neoplasia plus presence of HPV (n = 7) (1.9%). Neoplastic lesions of various degrees were presented by 43 patients (11.9%). Cytologicalcolposcopic-histological diagnostic procedures were used to confirm normal results, and histological diagnostic procedures were used to confirm lesions (Figure 4). 

## DISCUSSION

Debate continues regarding what the single strategy for optimal management of patients with ASCUS cytological reports might be.^1^ ^3 ^In our study, the population affected by this uncertain situation had a wide age range (17-88 years), which exceeded the limits (25-64 years) of the National Cervical Cancer Program. However, our age ranges were similar to those in Iraq, the United States and Turkey, in that the ages of females with a first atypical Pap result go from adolescence to adulthood.^17^ ^19^

The clinical management strategies for these women, i.e. those for whom referral to specialists or a Cervical Pathology Unit for first atypical Pap results is proposed by the algorithm, in accordance with the Bethesda 2001 classification, are outlined in the National Clinical Guidelines.^6^ At the diagnosis stage, the ASCUS cytological report is repeated, as indicated in our National Guidelines. This is also highly recommendable and advisable according to the various clinical guidelines of some other countries such as the United States, France and England.^1^ ^12^ ^13^ ^20 ^At this stage in Chile, diagnoses of HPV are not taken into consideration. In conformity with the standards established for the diagnosis stage, most of the women with first ASCUS cytological reports underwent a new Pap test six months later, in accordance with the clinical guideline regulation. Only 4.9% (18/363 smears) had the same atypical result. These data are similar to those found in the study by Tewari et al., in which 604 ASCUS cytological reports were repeated over a six-month period and the same results were only obtained in 3.8% of the smears.^21^ In Chile, Fanny et al. carried out an epidemiological study on 154 women with ASCUS reports and concluded that the appropriate length of time necessary before having the exam repeated is 6.4 months. With this time interval, outcomes of normal results or lesions were demonstrated.^22^

In our study, we found that 85.1% of the patients achieved normal results (n = 301), which was similar to the outcome of Tewari et al., in which the rate of regression to normalcy was 73.3%.^21^ The standard to follow according to what is indicated by the primary atypical Pap algorithm, is for patients to return to their PHC to continue with regular screenings, as established by the National Program for Research and Control of Cervical Cancer in Chile. 

**Figure:**
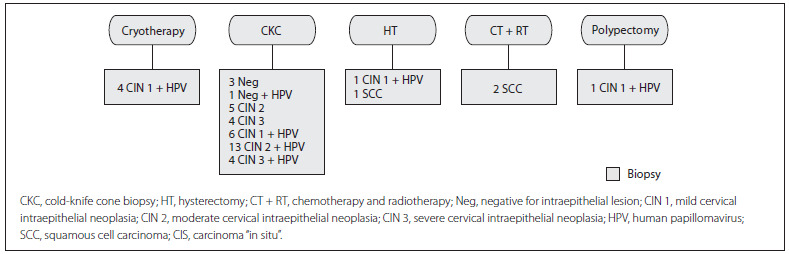
3. Treatment stage: according to the histological result.

Alternatively, abnormal results (n = 62) were sent to the CPU to continue with completion of colposcopy, which is a procedure established in the national algorithm. This decision is similar to the "acceptable" conduct advocated by the American Society for Colposcopy and Cervical Pathology, in which it is recommended to "repeat the Pap smear and colposcopy only as a reference if the second result is ASCUS+."^1^ ^23^ Following the aforementioned standard, 53 of our patients immediately underwent this examination: patients who met the cytological condition of ASCUS+ (30 low-grade lesions and 23 high-grade) and another nine women with clinical symptoms. Fulfillment of this colposcopic procedure was suggested by Kabaca et al., who recommended an aggressive conduct of immediate colposcopy when a first atypical Pap was presented. They conducted a study involving 205 female with ASCUS Pap smear results, 9.75% of whom presented CIN 2+ lesions.^20^ However, other studies have indicated that immediate colposcopy should only be performed on HSIL+ lesions, given that minor lesions return spontaneously.^24^ We recommend that colposcopy should only be performed when there are two consecutive atypical Pap tests, because with these two results, we were able to demonstrate 85.5% of the lesions, whereas colposcopy performed immediately after the first atypical Pap test only showed 16.9% of the lesions. 

An exploratory colposcopy examination is considered, within the algorithm, to be a procedure that would allow a decision to carry out a directed biopsy.^6^ Although the standards do not establish any necessity for a biopsy in cases of negative results, this was carried out in the majority of our cases (9 out of 13) and yielded two cases of a minor lesion (CIN 1). In cases of inconclusive results, a biopsy was also performed, yielding the following: 

Neg (n = 2) and CIN 2 (n = 1). 

The diagnostic confirmation stage was followed by the treatment stage, which was conducted in accordance with the literature.

Lastly, the follow-up was conducted on 363 cases in our cohort of women with a first ASCUS report. The 83 cases that were lost from the follow-up were not taken into consideration. The women who returned for follow-up evaluation presented a LSIL rate of 3.6% and a HSIL rate of 11.6%, both of which are within the range of results from other studies over the last five years, from 0% to 47.1% for LSIL and 0.8% to 8.6% for HSIL.^17^ ^18^ ^25^ ^26 ^It is clear that the percentage of HSIL lesions was higher than in the literature, but the cause is hard to explain. The diagnostic confirmation of these lesions was achieved by completion of a biopsy. This procedure was the same as has been established in the scientific literature, which considers that the efficiency of cervical cytological screening is determined through histological verification.^19^

These procedures that have been established through algorithms possess strengths and weaknesses. We consider that their strength lies in the contribution that they have made towards resultant lower rates of cervical cancer mortality in Chile. We consider that the lack of classification of the HPV virus is a weakness, given that there are no studies to support the algorithm regarding HPV. 

These algorithm-guided behaviors have contributed towards lower rates of cervical cancer mortality in Chile, which are among the lowest in Latin America.^27^ ^28^

## CONCLUSIONS

The clinical management observed through our monitoring shows that cases of a first ASCUS cytological report are managed in a traditional manner, in that it takes into consideration screening, diagnosis, diagnostic confirmation, treatment and posttreatment evaluation stages.

We agree with repetition of the first atypical Pap test six months later because this enables definition of the results.

The rate of regression to normality among the ASCUS cases was 85.1%, which confirms that Pap tests should only be repeated as part of the surveillance pattern.

We agree that women should be sent for immediate colposcopy whenever there are two consecutive atypical results, because of the high percentage of lesions (85.5%).
